# Studying the Effects of Alcohol Advertising on Consumption

**Published:** 1996

**Authors:** Henry Saffer

**Affiliations:** Henry Saffer, Ph.D., is a research associate at the National Bureau of Economic Research, New York, New York

**Keywords:** advertising, alcoholic beverage, AOD consumption, AOD abuse, economic theory, prevention strategy, public health, legal regulation, cigarette, marketing, communication media

## Abstract

The effects of advertising on alcohol consumption (and alcohol abuse) are controversial, and research on the subject has produced mixed results. An economic theory underlying the general relationship between advertising and consumption can help explain this variance, however. Studies that use national data on annual alcohol advertising expenditures measure advertising at a high level with little yearly change and are likely to find no effect on consumption. In contrast, studies that use local-level data measured over the course of a year find wide variation in the level of advertising and are likely to conclude that alcohol advertising significantly increases alcohol consumption. To mitigate consumption increases, some countries and localities have tested alcohol advertising bans or counter advertising campaigns. Studies of advertising bans show a decrease in alcohol consumption to some degree when intervening factors are controlled. Counter advertising likewise reduces alcohol consumption. Thus, policymakers can choose from various forms and combinations of these strategies to curb consumption and, presumably, alcohol abuse.

Alcohol advertising is a public health problem if it increases alcohol abuse. Yet despite considerable and well-documented levels of both alcohol advertising and alcohol abuse, the link between the two remains controversial. According to Leading National Advertisers (LNA) (1995), the alcoholic beverage industry spent more than $1 billion in 1994 for traditional media advertising (i.e., broadcast and cable television, radio, magazines, billboards, and newspapers). In addition, *The Economist* (1990) estimates that the industry annually spends a roughly equal amount on other forms of promotion, such as store displays, consumer novelties, and sponsorships of cultural and sports events. Likewise, the level of alcohol abuse is substantial. The National Institute on Alcohol Abuse and Alcoholism (NIAAA) estimates that in 1992, 14 million Americans met the diagnostic criteria for alcohol abuse or dependence^1^ (Grant et al. 1994). The Institute also estimates that in 1990 approximately 100,000 alcohol-related deaths took place, with roughly one-fourth of those deaths occurring on the highways (NIAAA 1993).

Does alcohol advertising increase alcohol abuse? The alcohol industry contends that advertising only induces consumers to switch from one brand of alcoholic beverage to another. In contrast, public health advocates assert that alcohol advertising increases total alcohol consumption and alcohol abuse. The challenge for researchers is to elucidate the true relationship between alcohol advertising and consumption and help resolve this controversy by providing solid data and well-grounded analyses.

Most studies on alcohol advertising examine the effect advertising has on alcohol *consumption*, measured in terms of either taxable withdrawals^2^ or self-reported consumption. Although alcohol *abuse*, rather than alcohol consumption, is the public health issue of specific concern, several researchers (for example, Schmidt and Popham 1978) have concluded that a proportionate relationship exists between average and abusive alcohol consumption and that this relationship is consistent for different populations. Accordingly, as average alcohol consumption increases in a population, abusive alcohol consumption can be expected to increase to the same degree. Following this reasoning, researchers consider average consumption to be a good proxy measure for alcohol abuse.

To examine the effects of alcohol advertising on alcohol consumption, researchers have turned to a type of statistical analysis called econometrics. Econometric studies attempt to determine how one economic variable (i.e., the dependent variable) responds to changes in other (i.e., independent) economic variables. In econometric studies of alcohol consumption, the dependent variable is alcohol consumption, and the independent variables include alcohol price, other relevant prices, consumer income, alcohol advertising, and other factors. All of these factors can affect alcohol consumption, according to a core economics concept referred to as “demand theory.”^3^

## Types of Econometric Studies

Researchers use four approaches to estimate the effect of alcohol advertising on total alcohol consumption. These four approaches consist of studies focusing on the following areas: (1) advertising measured by annual national expenditures, (2) advertising measured at the local level, (3) alcohol advertising bans, and (4) counter advertising efforts.

Some econometric studies have concluded that alcohol advertising has no effect on consumption, whereas a smaller group of studies have found that advertising does increase alcohol use and, presumably, alcohol abuse. To interpret these varied results, this article reviews econometric work on alcohol advertising and alcohol consumption (organized by approach) and places the studies in the context of an overall relationship between advertising and consumption. This relationship, called an advertising response function, basically shows that consumption increases as advertising levels rise, although the *rate* of increase in consumption eventually tapers off as a result of diminishing marginal product (see accompanying sidebar).

**Advertising Response Function: A Framework for Understanding Advertising and Consumption**

An economic theory underlying alcohol advertising and alcohol consumption provides some important insights into how econometric studies of alcohol advertising should be conducted and why certain econometric approaches produce results apparently at odds with other approaches. This theory posits that phenomena known as advertising response functions can describe the relationship between alcohol advertising and alcohol consumption. Advertising response functions have been used as the basis for research on brands of various products—from soda to saltines—to illustrate the effect that different levels of advertising have on product consumption.^1^

The advertising response function, which is a way of quantifying the relationship between advertising and consumption, is based on the theory of diminishing marginal product (see figure). Research shows that advertising “works” (i.e., consumption rises as advertising increases), but the economic theory of diminishing marginal product suggests that the relationship will level off at some point. According to the theory of diminishing marginal product, the continued addition of advertising messages eventually will lead to smaller and smaller increments of consumption. For example, suppose that adding 1,000 advertising messages per week to an existing advertising program of 5,000 messages per week will increase sales by 100 units per week. The theory of diminishing marginal product predicts that adding more advertising messages will not continue to boost sales by the same rate: The same 1,000 messages per week added to an advertising program of 50,000 messages per week may increase sales only by, say, 10 units per week.

Empirical work on advertisements of specific brands of beer (i.e., brand-level research) clearly supports the diminishing marginal product assumption (Rao and Miller 1975; Ackoff and Emshoff 1975). In the same way, the economic theory that describes the brand-level advertising response function can apply to a product-level response function—in this case, the product is defined as all alcoholic beverages. (The product-level response function does differ from the brand-level response function in one important way, however: Advertising-induced sales come at the expense of sales of nonalcoholic products instead of simply another brand of alcoholic beverage.)

To temper increases in consumption resulting from alcohol advertising, some countries and localities have adopted various forms of alcohol advertising bans. Generally, when alcohol advertising is banned from certain media, consumption still rises as advertising increases, but to a lesser extent than it would without the ban. The degree of reduction depends on the extent of the ban and the industry’s reaction to it. Although an advertising ban eliminates the use of specific media, it does not eliminate advertising.

The response to a ban can be seen as a three-step process. First, the alcohol advertisers will shift their existing advertising budget into the nonbanned media. Second, each of the nonbanned media are also subject to diminishing marginal product. As the existing budget is placed in the remaining media, the new consumption induced by this budget will be lower than it was before the ban. Third, in response to the decrease in effectiveness, the industry may increase or decrease its advertising in favor of alternative marketing strategies. For example, although a ban reduces the cost-effectiveness of advertising, the industry nevertheless may choose to expand total advertising efforts to regain the same level of sales as before the ban. Alternatively, the industry may decrease its advertising, because the cost per unit to induce sales may be prohibitive and other strategies, such as discount coupons, may be more cost-effective. In addition, a firm’s expectations about the advertising behavior of its rivals will influence its advertising decisions, further complicating predictions of advertisers’ response to a ban.

**Figure:**
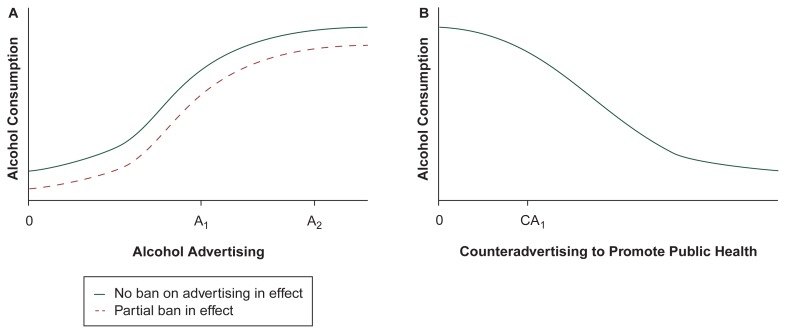
An advertising response function (A), with and without an advertising ban in place, shows the effect of alcohol advertising on alcohol consumption. It also shows the effect of elimination of a ban or addition of new media. Studies using national data on annual advertising expenditures (which vary little from year to year) measure advertising levels in a narrow range around A_2_, where advertising levels are high and the function is relatively flat. These studies generally find that small changes in advertising levels have little effect on consumption. In contrast, studies using local, short-term advertising data (which vary widely) measure advertising between 0 and A_1_, where the response function rises relatively steeply. These studies often find that increases in advertising raise consumption levels. A ban on alcohol advertising in certain media shifts the entire advertising response function downward, reducing consumption. A counter advertising response function (B) shows the effect that messages promoting public health have on alcohol consumption. Studies on counter advertising generally use data in the range between 0 and CA_1_, because the level of counter advertising is relatively low compared with the level of alcohol advertising. Because the function slopes downward fairly steeply in this range, these studies typically find that increases in counter advertising decrease alcohol consumption

The process can also work in reverse when new media become available. First, the existing advertising budget can be spent in more media. Second, all media are subject to diminishing marginal product. As the existing budget is placed in more media, the new consumption induced by this budget will be higher than it was before the new media became available. Third, in response to the increase in effectiveness, the industry may increase or decrease its advertising.

Advertising response functions also apply to counter advertising, which involves promoting public health through media messages (see figure). In the case of counter-advertising, however, consumption decreases as the level of counter-advertising rises. Because the law of diminishing marginal product also applies to counter advertising, this decrease in consumption will gradually taper off at high levels of counter advertising.

—*Henry Saffer*

ReferencesAckoffRLEmshoffJRAdvertising research at Anheuser-Busch, Inc. (1963–68)Sloan Management Review1621151975RaoRMillerPAdvertising/sales response functionsJournal of Advertising Research157151975SafferHAlcohol advertising and alcohol consumption: Econometric studiesMartinSEMailPThe Effects of the Mass Media on the Use and Abuse of AlcoholNational Institute on Alcohol Abuse and Alcoholism Research Monograph No. 28NIH Pub. No. 95–3743Bethesda, MDNational Institutes of Health19958399

1In addition to the advertising response function, two other important phenomena can influence the advertising-consumption relationship: reverse causality and cumulative advertising effects. Reverse causality is the idea that although advertising increases consumption, consumption also might increase advertising. Cumulative advertising effects occur when an advertisement’s initial impact lingers with a consumer and subsequent advertising messages build on each previous message. These issues and other econometric problems are discussed in more detail in Saffer (1995).

The following discussion also reviews econometric work on cigarette advertising and consumption. Cigarette studies involve the same econometric issues as alcohol studies do and provide further empirical evidence of the advertising response function. In addition, cigarette studies offer a unique opportunity to monitor the effects of counter advertising as a result of the Fairness Doctrine, a Federal initiative that mandated a level of counter advertising proportional to the level of cigarette advertising between 1967 and 1970. No comparable event has occurred in the history of alcohol advertising.

### Studies Using National Advertising Expenditure Data

Annual national alcohol advertising expenditures consist of the total of all alcohol advertising expenditures for all advertisers in all media and in all geographic market areas for an entire year. This total represents a high level of advertising that varies minimally from year to year. At this high level, the advertising response function predicts that studies are likely to find that small variations in the level of advertising have insignificant effects on consumption (i.e., no relationship exists between advertising and consumption).

A recent review of seven alcohol studies and six cigarette studies that employed annual national advertising expenditure data supports this prediction (see Saffer 1995 for further details). Three subsequently published alcohol advertising studies using annual national advertising expenditure data (Calfee and Scheraga 1994; Duffy 1995; Nelson and Moran 1995) offer additional support. Studies of this type typically analyze annual data drawn from a single country at 20 to 30 points in time and include control variables such as product price and consumer income.

### Studies Using Local-Level Measures of Advertising

For this type of study, researchers typically gather cross-sectional econometric data aggregated at a local level, such as from specific sets of cities and towns defined by the U.S. Census Bureau as Metropolitan Statistical Areas, for periods of less than 1 year. In contrast to annual national expenditure data, cross-sectional data (i.e., data collected at a particular point in time) are subject to wide variation. For example, advertisers may alternate between a high level of advertising and no advertising at all in specific localities (a practice known as pulsing) to keep the effectiveness of an advertising campaign from leveling off (i.e., to avoid diminishing marginal product). The pattern of these pulses fluctuates over different local areas. Because studies using local-level, cross-sectional data measure advertising over a range of high and low levels, the advertising response function predicts that they are likely to find that increases in advertising raise consumption levels (i.e., a positive relationship exists between advertising and consumption).

To date, only three relevant econometric studies have been conducted using cross-sectional data, but all three support the advertising response function. The first, a study of cigarette demand by Lewit and colleagues (1981), used a data set of more than 6,700 youth from several local areas. The youth were observed from 1966 to 1970, a period covering cigarette counter advertising required by the Fairness Doctrine, and data were collected on cigarette consumption as well as exposure to local advertising and counter advertising. The researchers found that advertising increased consumption while counter advertising had the opposite effect. The second study (Goel and Morey 1995) examined effects of both alcohol and cigarette advertising and used U.S. data on consumption organized by year (1959 to 1982) and State. Like Lewit and colleagues, Goel and Morey also found evidence that alcohol and cigarette advertising had positive and significant effects on consumption. A third study (Saffer in press) analyzed 1,200 quarterly aggregates of local-level data and examined the relationship between levels of alcohol advertising and highway fatalities over 4 years. Statistical analyses of advertising expenditures showed that alcohol advertising increases highway fatalities. Because more than 40 percent of highway fatalities are caused by drunk driving (National Highway Traffic Safety Administration 1993), this increase presumably reflects an increase in alcohol consumption levels as well.

### Studies of Alcohol Advertising Bans

An alcohol advertising ban usually eliminates advertising on broadcast television and radio, two media on which advertisers depend extensively because of the wide audiences they reach. For example, in the United States, 85 percent of all beer and wine advertising dollars are spent on broadcast media (LNA 1995).^4^ Enforcing a ban on broadcast television and radio compels advertisers to rely more heavily on other media outlets, such as billboard and magazine advertising (i.e., media substitution). Although a broadcast advertising ban does not necessarily decrease advertising expenditures, it does reduce the impact such expenditures have, because nonbanned media are subject to diminishing marginal product just as broadcast media are. Theoretically, an advertising ban will decrease consumption to the degree that the ban reduces the total effectiveness of the media left available for advertising. In addition, as more media are eliminated and media substitution becomes increasingly difficult for advertisers, the effect of a ban multiplies. Alternatively, if new media become available, the effectiveness of all media increases.^5^ (An analogy to an advertising ban might be forcing a given amount of traffic onto fewer roads. With fewer roads, traffic becomes congested, and all drivers are impeded. Likewise, as more advertising options are closed, the effectiveness of advertising expenditures is impeded.)

Because a ban diminishes advertising impact, econometric studies should find that advertising bans reduce alcohol consumption (i.e., have a negative effect) when comparing areas with and without a ban or comparing the same area before and after a ban. The seven studies conducted on alcohol and cigarette advertising bans have produced mixed results, however.

Three Canadian studies (Smart and Cutler 1976; Ogborne and Smart 1980; Makowsky and Whitehead 1991) examined the effect of alcohol advertising bans in the provinces of British Columbia, Manitoba, and Saskatchewan, respectively. In British Columbia, all alcohol advertising was abolished, but the ban lasted just 1 year. The ban in Manitoba covered beer advertising only and was analyzed over an 8-year period. In Saskatchewan, a 58-year-old ban on alcohol advertising was lifted. All three Canadian studies concluded that advertising bans had no effect on alcohol consumption. Although these results are contrary to the theoretical prediction, the econometric technique employed in the studies possibly could explain the discrepancy. In all three cases, the researchers used a technique known as interrupted time series, which tries to ascertain whether the variable of interest (e.g., alcohol consumption), measured over time, shifts when a particular policy (e.g., an advertising ban) is instituted. If so, then the policy is assumed to be the cause of the shift. This technique cannot account for other factors that may influence the variable, however (e.g., alcohol advertising crossing the U.S.–Canada border). The results of the Canadian studies also may indicate that research focusing on an advertising ban in a single province or country must collect data over a long period before any change in alcohol consumption can be observed.

Three studies of cigarette advertising bans using international data also have been published. The earliest study (Hamilton 1975) used data from 11 countries over the period 1948 to 1973 and compared countries with a ban to those without. Again, contrary to the expected result (i.e., that a ban would reduce consumption), the study did not find any significant effect of a ban. A second international study of cigarette advertising bans was conducted by Laugesen and Meads (1991) using data for the period 1960 to 1986 from 22 countries belonging to the Organization for Economic Cooperation and Development (OECD). (Member countries of the OECD have attempted to maintain a database of comparable economic and social data since 1960.) Like Hamilton, Laugesen and Meads found that cigarette advertising bans had no effect on consumption before 1973. After 1973, they found that cigarette advertising bans *did* have a significant negative effect on consumption. To explain this difference, Laugesen and Meads argue that before 1973, manufacturers were able to increase alternative marketing efforts (e.g., store displays, consumer novelties, and event sponsorships) in response to broadcast advertising restrictions. The study’s data set did not include measures of such marketing efforts, which could have offset the effect of the bans on broadcast advertising. The situation changed after 1973, when more comprehensive antismoking legislation enacted in several countries at about the same time restricted advertising efforts to a greater degree and resulted in lower cigarette consumption. The third and most recent study of cigarette advertising bans (Stewart 1993) analyzed data from 22 OECD countries for the period 1964 to 1990 and found that a television advertising ban had no effect. This study likewise did not control for other offsetting marketing efforts and, furthermore, did not separately examine data from the more restrictive period after 1973.

The seventh study in this category, a study of alcohol advertising bans (Saffer 1991), focused on data from 17 OECD countries for the years 1970 through 1983. This research compared indicators of alcohol abuse levels (i.e., average alcohol consumption, liver cirrhosis mortality, and motor vehicle fatality rates) in countries with and without an advertising ban. The results indicated that countries with bans on alcohol advertising generally had lower levels of alcohol abuse indicators, a finding that supports the theoretical prediction of a ban’s effectiveness.

### Studies of Counter advertising

The theory behind counter advertising is the inverse of the general advertising response function: As counter advertising increases, consumption levels decrease (i.e., a negative relationship exists between counter advertising and consumption). Although the level of counter advertising is relatively low compared with the level of alcohol advertising, studies that examine the effects of counter advertising campaigns are likely to agree with the theoretical prediction and find that increases in counter advertising do decrease consumption.

As mentioned previously, cigarette studies offer the best data for econometric studies on counter advertising because of the Fairness Doctrine initiative, as well as the antismoking publicity generated by the 1964 Surgeon General’s Report on Smoking. Several studies that measured the effect of cigarette counter advertising all concluded that counter advertising was effective in reducing cigarette consumption (see Saffer 1995 for a review). A comprehensive review by Flay (1987) of 56 counter advertising campaigns also concluded that counter advertising was effective in reducing cigarette consumption.

## Conclusions

Statistical evidence of the effect of alcohol advertising on alcohol consumption depends on the measuring stick used. Although studies of advertising’s effect on alcohol consumption have yielded inconsistent results, categorizing the studies according to the four types described in this article and putting them in the perspective of the advertising response function hold the key to explaining the differences.

Studies using the first type of approach (i.e., those using annual national expenditures as measures of advertising) are most common in the literature, because the data they use are relatively accessible, but these studies do not find an advertising effect. The other three types of studies, which are far fewer in number, primarily because of data limitations, do find effects of advertising, however. Integrating the results across all the studies gives the big picture: Although the total effect of advertising can be significant in terms of consumption level, the effect of small changes in advertising can be minimal in certain ranges (i.e., under the influence of the law of diminishing marginal product). Thus, extrapolating results from studies that measure advertising over a limited range of data may lead to erroneous conclusions. Overall, econometric studies appear to suggest that alcohol advertising increases alcohol consumption and counter advertising and advertising bans reduce alcohol consumption to some degree.

### Policy Implications

As noted previously, however, the public health issue at stake is alcohol abuse, not alcohol consumption. Assuming that alcohol consumption truly is a good proxy for alcohol abuse, econometric studies can be useful in determining which types of alcohol control policies may most effectively decrease alcohol abuse. Several policy options have been proposed, including limitations on advertising content, bans on the use of selected media, and taxation of advertising expenditures, as well as additional counter advertising.

Although limited, econometric studies to date suggest that either new restrictions on advertising or more counter advertising could help reduce levels of alcohol abuse. Therefore, policymakers can consider three general options among their choices: increasing both advertising restrictions and counter advertising together or increasing either one alone. The first option, increasing both advertising restrictions and counter advertising, may reduce alcohol abuse to the greatest degree, but Atkin (1993) argues that new limits on advertising would make it politically difficult to justify continuing or increasing the current level of counter advertising. A second option, increasing counter advertising without interfering with the industry’s freedom to advertise, could be funded by taxing alcohol advertising. Although a heightened counter advertising campaign probably would prompt more advertising by the industry, econometric studies and advertising response functions suggest that the effect of increased counter advertising may outweigh the effect of additional advertising. This option thus also would decrease alcohol abuse, but to a lesser extent than if expanded counter advertising were coupled with advertising restrictions. Econometric research implies that the third option—adding limits on advertising alone, without accompanying counter advertising campaigns—may also decrease alcohol abuse. This option probably would result in the least decline in alcohol abuse, however, because media substitution could partially offset the new restrictions.

Glossary

Advertising response function: The relationship between advertising and product consumption. Brand-level response functions describe the effect of advertising on consumption of particular product brands (e.g., Bud Lite^®^). Although brand-level response functions are most commonly studied, the concept also can be applied at a product level to describe the effect of advertising on consumption of a product class (e.g., all alcoholic beverages).
Demand theory: An economic theory that states that the willingness and ability of consumers to purchase a commodity (e.g., alcohol) can be influenced by factors such as the price of the commodity, other relevant prices, consumer income, and advertising messages.
Dependent variable: A variable whose value is determined by one or more *independent variables* in a function. In *econometric* studies of alcohol consumption, the level of consumption is the dependent variable.
Diminishing marginal product: An economic theory that states that beyond a certain point the *marginal product* will fail to change in proportion to additional inputs of labor or capital. Applied to advertising, this means that incremental advertising expenditures will result in incremental consumption, but the size of the increments in consumption diminishes as this process continues (i.e., increases in alcohol consumption will level off as alcohol advertising increases beyond a certain amount).
Econometrics: The application of statistical methods to the study of economic data.
Independent variable: A variable whose value determines that of the *dependent variable* in a function. In *econometric* studies of alcohol consumption, independent variables could include alcohol advertising, alcohol price, consumer income, and other factors.
Marginal product: In an economic function, the amount of change in output produced by an additional unit of input (e.g., the amount by which alcohol consumption levels rise in response to increased advertising).

## Footnotes

1 The definitions of “alcohol abuse” and “alcohol dependence” in this citation and throughout this article correspond to the diagnostic criteria set forth for these conditions in the American Psychiatric Association’s *Diagnostic and Statistical Manual of Mental Disorders, Fourth Edition*.

2 Alcoholic beverages are taxed when withdrawn from their U.S. manufacturer’s or importer’s inventories and shipped to distributors. These are known as taxable withdrawals. Taxable withdrawals are considered a good measure of consumption.

3 For a definition of this and other technical terms, refer to the glossary on p. 271.

4 No ban on alcohol advertising currently exists in the United States at the Federal level. Nevertheless, while beer and wine advertisements have proliferated on television and radio, liquor manufacturers have abided by a voluntary ban against broadcast advertising since 1948 until recently. In November 1996, liquor manufacturers formally dropped the voluntary ban on broadcast advertising and began airing advertisements for spirits on cable television and mainstream radio.

5 New media include cable TV, radio, and the World Wide Web. According to the Center for Media Education, alcohol advertising is increasing on the World Wide Web. Alcohol advertisers have created a number of Web sites that attract visitors in several ways, including friendly virtual bartenders and stories and interactive games which involve the product. Alcohol advertisers also pay search engine companies for key words and for prominent placement (e.g., placement in the first 10 hits on a key word).
